# Ewing's Sarcoma of the Talus in an Adolescent Female: An Unusual Case Presentation With Review of Literature

**DOI:** 10.7759/cureus.30946

**Published:** 2022-10-31

**Authors:** Divesh Jalan, Ravi Sreenivasan, Rohit Prasad, Dharmendra K Singh, Amit K Jha

**Affiliations:** 1 Orthopaedics, Vardhman Mahavir Medical College and Safdarjung Hospital, New Delhi, IND; 2 Radiology, Vardhman Mahavir Medical College and Safdarjung Hospital, New Delhi, IND

**Keywords:** metastasis, talus, ankle and foot, adolescent, ewing's sarcoma

## Abstract

Ewing’s sarcoma is an aggressive primary malignant bone tumor that affects long and flat bones and is commonly seen in children and adolescents. The involvement of the foot especially the talus is an extremely rare entity with less than 15 cases reported in the literature. The rarity and atypical symptoms often lead to delays in diagnosis affecting the prognosis and survival. We present a 14-year-old female with pain and swelling of the left ankle for 18 months. She was being treated previously for an ankle sprain and was later suspected of avascular necrosis of the talus, before presenting to us. Clinicoradiology-pathological workup confirmed the diagnosis of Ewing sarcoma of the talus. Further, the metastatic workup revealed multiple skeletal metastases at the time of diagnosis. The metastatic lesion of the right femur required prophylactic fixation, otherwise, the patient was treated with palliative chemotherapy and radiotherapy. Ewing’s sarcoma of the foot involving the talus is extremely rare and is a commonly misdiagnosed entity, affecting the overall prognosis of the patient. A high index of suspicion and a multidisciplinary approach is imperative for its early diagnosis and definitive management.

## Introduction

Ewing’s sarcoma (ES) is an aggressive primary malignant bone tumor that is commonly observed in the age group of less than 20 years [1]. It is the second most common type of primary bone cancer in children and adolescents and comprises around 15 % of all bone malignancies [2,3]. Around 20% of patients have metastases at the time of diagnosis [2].

Ewing’s sarcoma commonly involves the axial and appendicular skeleton but is rarely seen in the foot especially in the talus [4]. Although the first case of Ewing’s sarcoma in the talus was described in 1953 [5], less than 15 cases have been reported until now. The rarity of the disease and its atypical location in the foot often result in delays in the diagnosis and definitive treatment of such patients, affecting the overall prognosis of these patients.

## Case presentation

**Figure 1:**
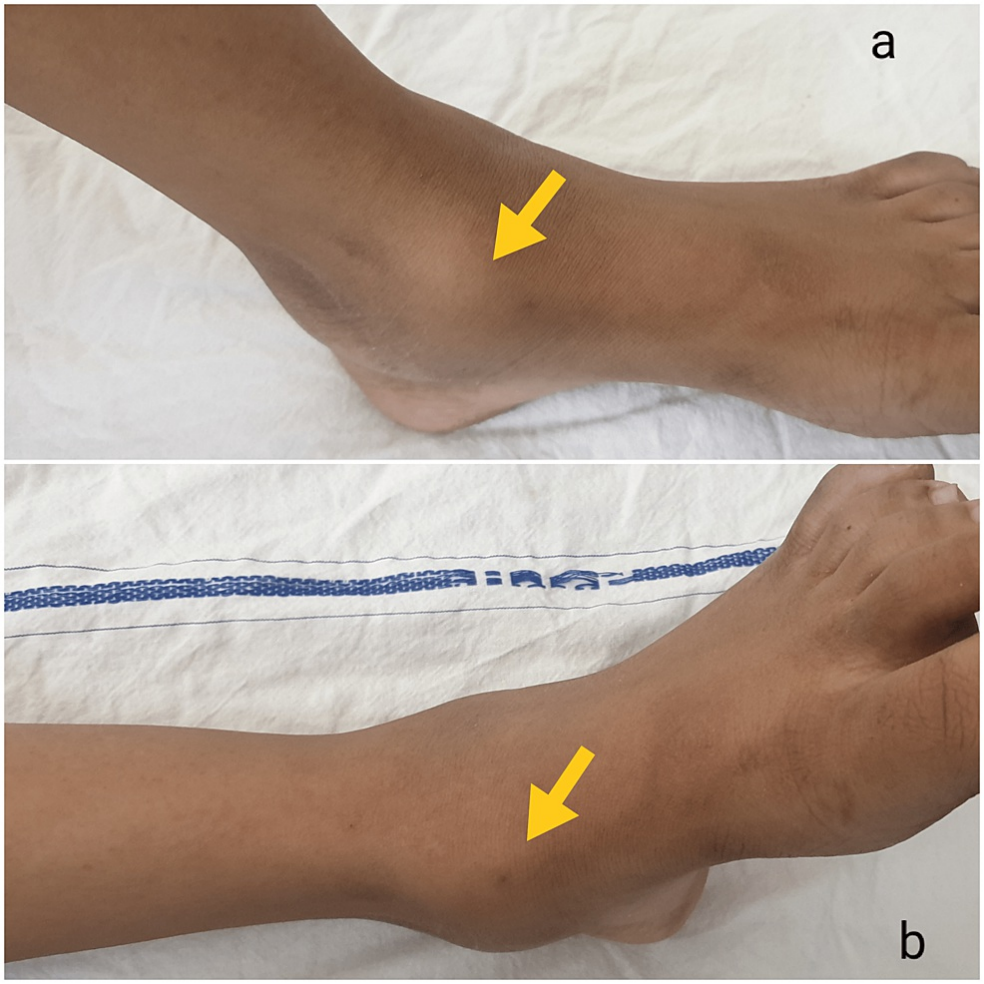
Photographs of the left ankle where image a and image b demonstrate an ill-defined, firm, tender swelling at the medial aspect of the ankle (yellow arrow).

A 14-year-old female presented to the outpatient clinic with chief complaints of pain and swelling around the left ankle for 18 months. She had a history of a twisted ankle 18 months ago, for which she was treated conservatively. However, the pain and swelling around the ankle gradually progressed and were not relieved completely by medications. Before presenting to us, she had taken treatment for an ankle sprain and subsequently was suspected of avascular necrosis of the talus. The pain was aggravated by strenuous activities and relieved on rest. It was not associated with constitutional symptoms of night cries, weight loss, fever, etc. There was no clinical involvement of other joints. The past and family history were irrelevant. On physical examination, an ill-defined, firm, tender swelling at the left ankle joint was noted (Figure 1).

**Figure 2:**
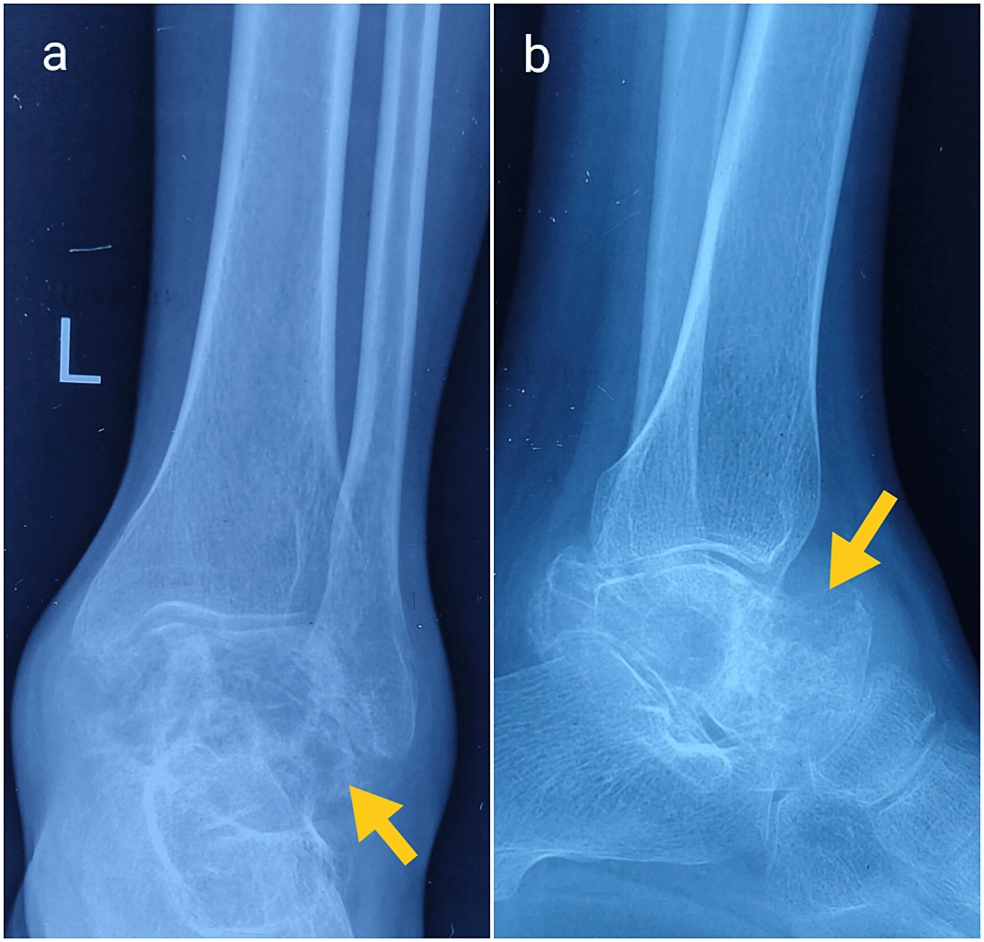
Radiographs of the left ankle The anteroposterior (a) and lateral (b) views demonstrate an osteolytic lesion involving the talus with cortical erosion and soft tissue swelling (yellow arrows).

The range of motion in the ankle joint was decreased and was terminally painful. The erythrocyte sedimentation rate was 65mm/hour while the total leukocyte counts were within the normal limit. The radiograph demonstrated an osteolytic lesion of the talus with collapse and cortical destruction, suggestive of the aggressive osseous lesion (Figure 2).

**Figure 3:**
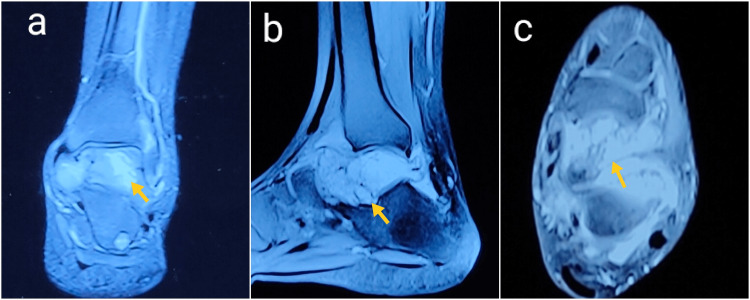
Fat-suppressed T2-weighted MRI The coronal (a), sagittal (b), and axial (c) views of the left ankle demonstrate hyperintense lesion in the talus (yellow arrow) with soft tissue extension and cortical destruction.

The patient was further evaluated with magnetic resonance imaging (MRI) of the ankle joint that demonstrated a heterogeneous lesion of the talus; predominantly hypointense on the T1-weighted image, and hyperintense on the T2-weighted image and fat saturation (FAT SAT) image with soft tissue extension and cortical destruction (Figure 3). Based on the clinico-radiological evaluation, a differential diagnosis of an aneurysmal bone cyst (ABC), giant cell tumor (GCT), or chronic osteomyelitis of the talus was suggested. 

**Figure 4:**
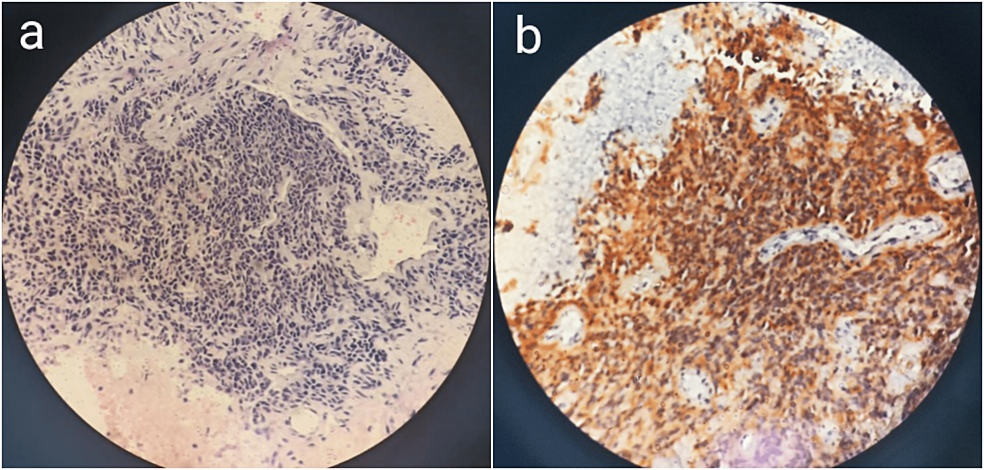
Histopathology photomicrographs (H&E stain 10x) (a) Sheets of small round cells with a high nuclear to cytoplasmic ratio, (b) The tumor cells show diffuse membranous expression of CD99 in IHC H&E: Hematoxylin and eosin, CD99: Cluster of differentiation 99, IHC: Immunohistochemistry

For histopathological diagnosis, an ultrasound-guided (USG) biopsy was performed which revealed a malignant round-cell tumor (Figure 4, a). Immunohistochemistry (IHC) stains were positive for cluster of differentiation (CD)99 (Figure 4, b), B-cell leukemia/lymphoma 2 (BCL2), and Friend leukemia integration 1 (FLI1) confirming the diagnosis of Ewing’s sarcoma.

**Figure 5:**
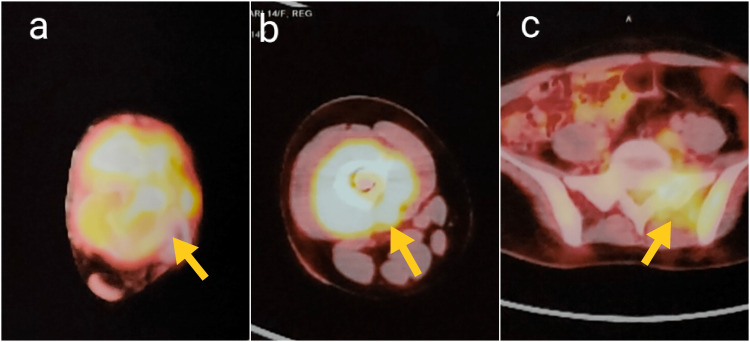
PET/CT images (a, b, c) demonstrate increased tracer uptake in the left talus, right shaft of the femur, and left sacrum and hemi pelvis (yellow arrows). PET/CT: Positron emission tomography/computed tomography

As the primary malignant tumor was not suspected clinicoradiologically, systemic staging was performed after the biopsy. The staging was done with whole body positron emission tomography (PET)/computed tomography (CT) scan with 18-fluoro-deoxyglucose (FDG) which revealed multiple skeletal metastases (right femur, sacrum, left hemipelvis, and right posterior fifth rib) (Figure 5).

**Figure 6:**
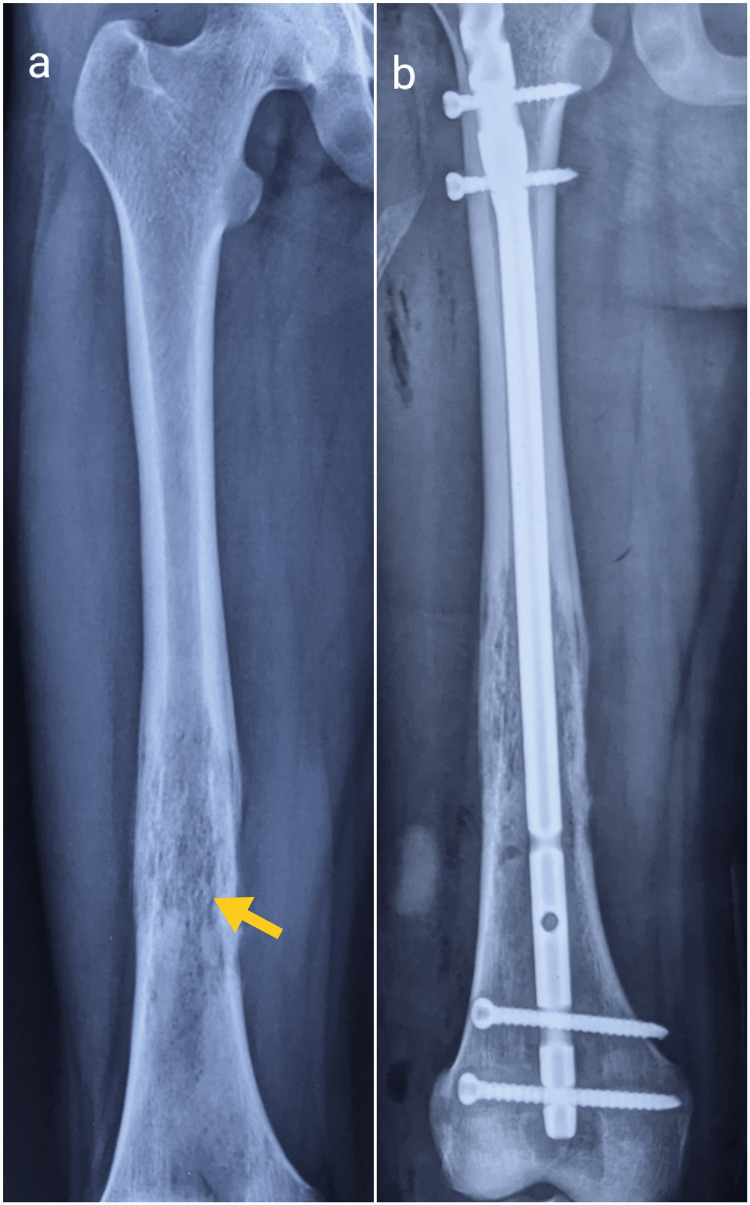
Radiographs of the right femur a: Permeative destruction in diaphysis (yellow arrow) suggestive of bone–to-bone metastases, b: Prophylactic intramedullary nailing done for the right femur metastases

The patient became symptomatic with pain in the right thigh during the course of diagnosis. A radiograph of the right femur was performed, which revealed permeative destruction with a periosteal reaction involving the femoral diaphysis (Figure 6, a). The Mirel’s score for impending fracture risk was 9 so, the decision for prophylactic reamed intramedullary interlocking nail (IMIL) was made (Figure 6, b).

The case was discussed in the institutional tumor board and, in view of extensive skeletal metastasis and a poor prognosis, it was decided to treat the patient with palliative chemotherapy and radiotherapy. Currently, the patient has received the fourth dose of chemotherapy and is under follow-up.

## Discussion

Ewing’s sarcoma is a small round-cell tumor that can affect any part of the skeleton. It is the second most common malignant bone tumor in children younger than 20 years. It shows a predilection for males with a ratio of 1.4 to 1. It usually arises from the diaphysis or meta-diaphyseal region of the long bones. Pelvic bones and ribs are also commonly affected. Less frequent and rare locations are the skull bones, scapula, vertebra, and the small bones of the hand and feet [6].

Foot involvement is seen only in 0.93% to 4.2% of ES, most commonly involving the calcaneum and metatarsals [1,7]. Talus involvement is seen very rarely, with only 13 cases reported until now (Table 1). The first case was reported by Cohen et al. in 1953 [5].

**Table 1:** Summary of case reports on Ewing's sarcoma of the talus NR: Not reported, RT: Radiation therapy, CT: Chemotherapy, M: Male, F: Female

S.no	Author / Year	Age / Sex	Symptoms	Duration (Month)	Metastasis	Treatment	Survival (Months)
1.	Cohen et al. [5], 1953	3yr/M	Painful limp of the left leg	30	Widespread dissemination		32
2.	Alpert et al. [8], 1965	8yr/M	Painful ankle	3		RT	45
3.	Weissman et al. [8], 1966	18yr/F	Sprained ankle	6		Deep RT	6
4.	Pandey et al. [8], 1970	16yr/F	Low-grade fever, pain, and swelling	3		Deep RT	4 & ½
5.	Shirley et al. [8], 1985	12yr/F	Pain & swelling	2		RT & CT	42 (alive)
6.	Maletz et al. [8], 1986	13yr/M	Pain & swelling	24	Lungs	CT & below knee (B/K) amputation	30 (alive)
7.	Adkins et al. [8], 1997	20yr/F	NR	NR	Lungs	RT & CT	8
8.	Serrano et al. [8], 1998	14yr/M	NR	NR	NR	CT	NR
9.	Rasit et al. [8], 2001	4yr/F	Osteomyelitis	8	Pleura & spine		8
10.	Kim et al. [8], 2004	29yr/M	Sprain	7		NR	48 (alive)
11.	Rammal et al. [9], 2008	10yr/M	Pain & swelling	1	Lung	CT & B/K amputation & RT	12 (alive)
12.	Salunke et al. [10], 2018	12/F	Pain & swelling	14		CT & B/K amputation	32 (alive)
13.	Panayiotis et al. [11], 2019	30yr/F	Pain	12		CT & limb salvage	41 (alive)
14.	Present case	14/F	Pain & swelling	18	Bony metastasis	CT	7 (alive)

Our review of the literature revealed a mean age of 14.4 years (range: three to 30 years), with a female-to-male ratio of 1.14:1. The most common symptom reported was ankle pain and swelling with a mean duration of symptoms of 10.76 months (range: two to 30 months). It was also noted that 42.8 % of the patient had distant metastasis at the time of presentation. Most of the patients were treated with radiation therapy &/or chemotherapy, while three patients underwent amputation below the knee and one patient underwent limb salvage.

The most common symptom is pain and swelling in the ankle and around 20% of patients have an associated history of trauma [12], which was seen in our case too. The majority of these patients suffer from protracted symptoms as they are commonly misdiagnosed and treated for other conditions such as soft tissue injuries, avascular necrosis, etc. [12]. Yang et al. [13] reported a median duration of 52 weeks in the delay in the final diagnosis from the onset of symptoms. In our case, this problem is again highlighted with a delay of almost 18 months.

The plain radiographs are often characterized by a ‘permeative’ or ‘moth-eaten’ pattern of a destructive osteolytic lesion, seen most commonly in long bones. Bone destruction is often more marked than periosteal reaction in the foot and ankle, and may have an atypical appearance that mimics a benign lesion, which was also seen in our case. The classical periosteal reaction ‘onion skin peel' is more often seen in diaphyseal tumors, as is evident in our case of right femur metastasis [14]. The lesion must be further evaluated with MRI, which is useful in assessing the extent of the tumor, characterization of the tumor, and its relation to surrounding neurovascular structures, and also helps to plan the target area of biopsy [14]. Systemic staging involves identifying distant metastasis. Nowadays, the most commonly performed investigation to assess metastasis is PET/CT, which helps detect both skeletal and visceral metastases and obviates the need for bone marrow biopsy [15]. Common metastatic sites include lung, bones, liver, brain, and distant lymph nodes [2]. In our case, extensive bony metastases were seen on PET/CT, of which the right femur required prophylactic fixation.

The gold standard of the diagnosis is histopathology, which requires a biopsy. Although open biopsy is considered the gold standard procedure, core needle biopsy being minimally invasive provides adequate tissue in the majority of cases. Therefore, open biopsy is only required when repeated attempts at obtaining adequate tissue with core biopsy have failed. In our case, an ultrasound-guided core needle biopsy was performed and sent for histopathological evaluation. Microscopically, it is composed of sheets of small round blue cells with a high nuclear-to-cytoplasmic ratio [6]. Ewing's sarcoma falls into the ‘small round cell’ tumor group and similar findings are observed in several other tumors such as small cell osteosarcoma, mesenchymal chondrosarcoma, lymphoma, etc. Additional investigations including IHC markers and molecular genetics help in narrowing the differentials and making the final diagnosis. The CD99, an IHC marker, is positive in more than 90% of cases of ES but can be seen in several other conditions such as lymphoblastic lymphoma, small cell osteosarcoma, mesenchymal chondrosarcoma, etc. For this reason, FLI-1 immunoreactivity in a suspected primary small round cell bone tumor strongly favors the diagnosis of ES [16]. In our case, the diagnosis of ES was made as the patient showed classical histological findings and IHC markers showed positivity for CD99 and FLI-1.

Treatment of ES depends on the stage of the disease; multimodal treatment involving radiotherapy or surgery for local control and chemotherapy for systemic control is usually recommended [10]. In cases involving the foot, where there is no metastasis, amputation is preferred over limb salvage [13,17]. The reason includes poor tumor compartmentalization in the foot, which makes adequate resection difficult, and problems with bony and soft tissue reconstruction. Metastasis at presentation is observed in almost 20% to 25% of patients, most commonly in the lung followed by skeletal metastasis [9,13]. In our case, the systemic staging revealed multiple skeletal metastases out of which one of the lesions in the shaft of the femur became symptomatic during the course of management.

Ewing’s sarcoma has the worst prognosis and has the shortest median time to death compared with any other osseous foot malignancy [13]. Patients with metastatic ES at presentation fare much poorly and thus should be treated with chemotherapy and radiation without surgery [18]. The five-year survival of patients with localized disease is significantly higher (~80%) compared to patients with bony metastasis (~30% to 19%) [19]. Because of the poor prognosis in our case, the decision of palliative chemotherapy and radiotherapy was taken by the tumor board. 

## Conclusions

Ewing’s sarcoma of the talus is extremely rare which poses challenges in the diagnosis because of its atypical location and non-specific symptoms. Delay in diagnosis often compromises treatment due to metastases and results in a poor prognosis in these patients. Therefore, a high index of suspicion is required in young children with chronic symptoms and aggressive osseous lesions on imaging. They should be thoroughly and timely evaluated to achieve a better outcome.
